# Marble Waste Sludges as Effective Nanomaterials for Cu (II) Adsorption in Aqueous Media

**DOI:** 10.3390/nano11092305

**Published:** 2021-09-05

**Authors:** Ventura Castillo Ramos, José Rivera Utrilla, Antonio Ruiz Sánchez, María Victoria López Ramón, Manuel Sánchez Polo

**Affiliations:** 1 Department of Inorganic Chemistry, Faculty of Science, University of Granada, 18071 Granada, Spain; jrivera@ugr.es (J.R.U.); mansanch@ugr.es (M.S.P.); 2 Department of Structure Mechanics and Hydraulic Engineering, University of Granada, 18071 Granada, Spain; antonioruiz@ugr.es; 3 Department of Inorganic and Organic Chemistry, Faculty of Experimental Science, University of Jaén, 23071 Jaén, Spain; mvlro@ujaen.es

**Keywords:** marble waste sludge, copper, adsorption, water remediation, kinetics, isotherms

## Abstract

This study evaluated the waste generated by a Spanish marble-producing company as adsorbent for the removal of copper (Cu [II]) from aqueous media. Six marble waste sludge samples were studied, and the following operational parameters were analyzed in discontinuous regime, including pollutant concentration, pH, temperature, nature of aqueous medium, and ionic strength. The applicability of the adsorbent material was assessed with experiments in both continuous and discontinuous regimes under close-to-real-life conditions. A pseudo-second order model yielded a better fit to the kinetic data. Application of the intraparticle diffusion model revealed two well-differentiated adsorption stages, in which the external material transfer is negligible and intraparticle diffusion is the controlling stage. The equilibrium study was better fitted to a Freundlich-type isotherm, predicting elevated maximum adsorption values (22.7 mg g^−1^) at a relatively low initial Cu (II) concentration (25 ppm), yielding a highly favorable chemisorption process (n >> 1). X-ray fluorescence study identified calcite (CaCO_3_) as the main component of marble waste sludges. According to X-ray diffraction analysis, Cu (II) ion adsorption occurred by intercalation of the metallic cation between CaCO_3_ layers and by the formation of surface complexes such as CaCO_3_ and Cu_2_(CO_3_)(OH)_2_. Cu (II) was more effectively removed at medium pH, lower temperature, and lower ionic strength of the aqueous medium. The salinity and dissolved organic matter in surface, ground-, and waste-waters negatively affected the Cu (II) removal process in both continuous and discontinuous regimes by competing for active adsorption sites. These findings demonstrate the applicability and effectiveness of marble-derived waste sludges as low-cost and readily available adsorbents for the treatment of waters polluted by Cu (II) under close-to-real-life conditions.

## 1. Introduction

Pollution associated with industrial development has been investigated over the past few decades. Pollutant emissions can have adverse effects on plants, animals, and humans as a consequence of long-term contact with or frequent exposure to concentrations in air, water, and ground. Specifically, aqueous emissions of copper (Cu [II]) can have severe consequences for health and the environment, being toxic for aquatic ecosystems and bio-accumulative, and it can even disrupt nutrient cycling processes [1]. Most Cu (II) waste comes from burning fossil fuels in energy- and metal-producing plants and from waste incinerators, wastewater treatment plants, and pesticides. One of the main sources of environmental pressure in Europe is the discharge of Cu (II) mining waste into the water [2]. Given its elevated toxicity, the World Health Organization (WHO) has established a maximum Cu (II) concentration of 2 mg L^−1^ in waters intended for human consumption [3] and the United States Environmental Protection Agency a maximum concentration of 1.3 mg L^−1^ [4,5]. Solutions are urgently needed for the effective removal of heavy metals from water, including Cu (II).

Heavy metals can be removed from water by chemical precipitation [6], ion exchange [7], filtration [8], and/or electrochemical treatments [9]. However, their high cost and energy requirements make them unattractive for direct application in real-life processes [10]. Adsorption is the most frequently applied method to remove heavy metals from waters [11], air [12,13], and soil [14], and activated carbon is the most widely used adsorbent. The high adsorption power of activated carbon is attributable to its large and varied pore network, among other characteristics [15,16,17].

There has been a marked intensification of efforts to develop low-cost adsorption processes and materials with increased metal uptake capacity and reduced environmental impact. Low-cost materials can include those that are locally available in large amounts, such as mineral components, agricultural waste, or industrial by-products [18,19]. Low-cost materials used to date include fly ash, silica gel, chitosan, agricultural waste, wood residues, dolomite, and clays [18,20,21].

Various authors have investigated the heavy metal adsorption capacity of marble industry waste, including sludges, powders, and derivatives (Table 1). Alwared et al. [22] used dry marble powder for competitive removal by flotation and achieved the removal of 99.95% of Pb (II), 84.58% of Cu (II), and 78.70% of Cd (II) ions within a relatively short time period. Wazwaz et al. [23] tested the adsorption of different heavy metals using soils, pure marble, impure marble (sludge from marble factories), and a marble and granite mixture, reporting Cu (II) adsorption of 91.40% for marble sludge versus 92.29% for pure marble. Javed et al. [24] synthesized a zeolite from marble powder and obtained higher Cu (II) adsorption (99.29%) than achieved with natural zeolite. Tozsin [25] used marble cutting waste and removed >80% by weight of Cd, Cr, Cu, Ni, Pb, and Zn from the acid drainage of mines generated by Cu (II) flotation tailings.

Marble waste, especially marble sludge, represents 30–50% of the final product volume, and large amounts are generated in China, Spain, Italy, and Brazil, among other countries [26,27]. The volume of this waste is too high to be feasibly stored, and it is generally released by marble industries into the environment in a controlled or uncontrolled manner [27,28]. The effective reutilization of this waste not only yields economic benefits but is also crucial to reduce environmental pollution.

With this background, this study investigated the reutilization of marble sludge generated by a local Spanish company (Macael S.I) in the removal of Cu (II) from polluted waters. The objectives were: to study the adsorption process by kinetic and equilibrium analyses in static and dynamic regimes; to evaluate the influence of the temperature, pH, ionic strength, and chemical composition of the water; to describe the adsorption mechanism, and to explore the applicability of this waste.

## 2. Materials and Methods

This study used marble waste sludge from six ornamental stone processing industries in Macael (Almeria, Spain). Waste sludge was directly used in the experiments with no pretreatment or chemical modification. Solutions in static adsorption experiments were prepared using ultrapure water (Milli-Q equipment, Millipore, Darmstadt, Germany), wastewater, surface water, and groundwater samples that were contaminated by adding CuSO_4_ (Sigma Aldrich, 99.99%, Darmstadt, Germany) at the studied concentrations. NaNO_3_ (99.99%), HCl (37%), and NaOH pellets were supplied by Sigma Aldrich and used without any pretreatment. The same types of water sample and chemicals were used in the dynamic adsorption experiments.

### 2.1. Characterizations

A scanning electron microscope (SEM) (HITACHI S-510, Austin, TX, USA) was used for morphological and elemental analyses of powdered waste, depositing samples on sample holders and metalizing them with carbon; the voltage was set at 20 kW and working distance at 22–25 mm.

The chemical composition of samples was examined by using a sequential wavelength dispersive X-ray spectrometer with a power of 4 kW (PHILIPS MAGIX PRO, PW-2440, Austin, TX, USA). Samples were prepared as melted disks (pearls) using a mixture of 50% lithium metal and tetraborate as flux. An automatic pearler (Model PERLX3, PHILIPS, Kissimmee, FL, USA) was used when a sufficient amount of sample was available; if not, pearls were manually prepared.

The mineralogical composition of the samples was qualitatively analyzed by X-ray diffraction (XRD) using a BRUKER D8 ADVANCE (Madrid, Spain) powder diffractometer equipped with a 4 kW high-voltage generator and X-ray tube with Cu anode operating at 40 kV and 30 mA. Diffractograms were always recorded in the interval between 3° and 80° 2θ with a passage size of 0.02°. Crystalline substances were identified using DIFRACCPLUS BASIC and the EVA package of BRUKER and were compared with the catalogued pattern table. Before analysis by XRD, samples were ground and sieved to below 45 μm to obtain a larger amount of particles and a random distribution.

The granulometric distribution of the studied particles was determined by using a laser diffractometer MASTERSIZER model 2000LF (Malvern Instruments Ltd., Madrid, Spain) on samples in liquid suspension with a solution of 1.8 g sodium hexametaphosphate and 0.4 g anhydride sodium carbonate in 1 L of water. Samples were then subjected to ultrasound action for 5 min and left to rest for at least 24 h to achieve the total dispersion of particles. Next, samples were again subjected to ultrasound and magnetic agitation before being placed in the measurement tray of the equipment.

### 2.2. Batch Adsorption Tests

Adsorption experiments were conducted in static regime to determine the adsorption kinetics, corresponding isotherms, and the influence of the pH, ionic strength, and chemical composition of the water. These experiments were prepared by adding 0.1 g adsorbent in 100 mL solution with initial Cu (II) concentrations ranging between 4.5 and 45 mg L^−1^. The temperature was controlled by using a water bath with a precision of ±0.1 °C, maintaining constant agitation at 200 rpm. Equilibrium was always reached at around 50 h. The Cu (II) ion concentration was determined by reverse-phase high-performance liquid chromatography (HPLC) using a liquid chromatograph (Thermo-Fisher, Santa Clara, CA, USA) equipped with a UV-detector and autosampler with capacity for 120, taking aliquots of solution at specific time points and filtering them before measurement of the concentration.

The amount of Cu adsorbed on the adsorbent was calculated using Equation (1).

(1) $$Q=\frac{C_{0}-C}{m}\;V$$

where Q represents the amount of pollutant adsorbed in equilibrium, in mg g^−1^; V is the volume of solution, in L; m is the adsorbent mass, in mg; C_0_ and C are the Cu (II) concentrations in the initial solution and at equilibrium, respectively, in mg L^−1^.

The percentage Cu (II) removed (*R*) was calculated using Equation (2).

(2) $$R=\frac{C_{0}-C}{C_{0}}\;100$$

The applicability of the material for treating waters polluted with Cu (II) was examined by analyzing the effects of operational variables. The influence of pH on Cu (II) adsorption was tested by preparing solutions of Cu (II) (25 mg L^−1^) in ultrapure water at pH values ranging between 2 and 8, which were adjusted by HCl and NaOH solutions and obtained by direct measurement with a pH meter (Hach Sension^+^ PH 3). The influence of temperature was studied at 20, 50, 75, and 90 °C using solutions of 25 mg L^−1^ Cu (II) prepared in ultrapure water at pH 5. The effect of the ionic strength of the solution was evaluated by performing six adsorption experiments in static regime and adding NaNO_3_ to a solution with an initial concentration of Cu (II) (25 mg L^−1^), temperature of 25 °C, pH of 5, and 0.1 g of adsorbent. The influence of the chemical composition of the water was explored by comparing among surface waters, wastewaters, and groundwaters, measuring concentrations of [HCO_3_^−^] ions and total organic content (TOC).

### 2.3. Dinamic Adsorption Tests

Dynamic adsorption experiments were conducted in columns with a diameter of 1 cm and height of 10 cm that were loaded with marble waste sludge. A Cu (II) solution was then passed through columns with a peristaltic pump, always at a concentration of 100 mg L^−1^, flow of 1.5 mL min^−1^, and temperature of 25 °C (in thermostatic bath). Experimental breakthrough curves were constructed, and the following parameters were obtained: X_0.02_ (amount adsorbed at breakthrough point); V_0.02_ (breakthrough point volume); Φ (fractional capacity); HMTZ (height of mass transfer zone), and Du (degree of utility), following the models described elsewhere [33]. The same HPLC equipment as for batch adsorptions was used to determine the concentration of Cu (II) ions in the effluent.

## 3. Results

### 3.1. Characterizations

Characterization of the marble waste sludge samples showed that the friction between ironwork and boulders and slabs of marble generates an extremely thin grain-sized material with no structure. Figure S1a depicts a typical SEM image of a sludge sample. Granulometric analysis of the materials confirmed their very small particle size, even smaller than that of clays, with around 70% of particles having a maximum size smaller than one micron (Figure S1b).

X-ray fluorescence results indicate that the main component was CaO (55%) in all sludge samples, and that no other component reached 1% of the total mass of samples, observing only trace amounts (Table 2).

### 3.2. Batch Adsorption Tests

All six marble waste sludge samples showed very similar Cu (II) adsorption behavior and performance with virtually identical profiles (Figures S2 and S3). Therefore, the six samples were grouped together for analyses of the results of adsorption and equilibrium kinetic studies, including error bars to represent the standard deviation in results among individual samples.

#### 3.2.1. Kinetics Study

**Figure 1:**
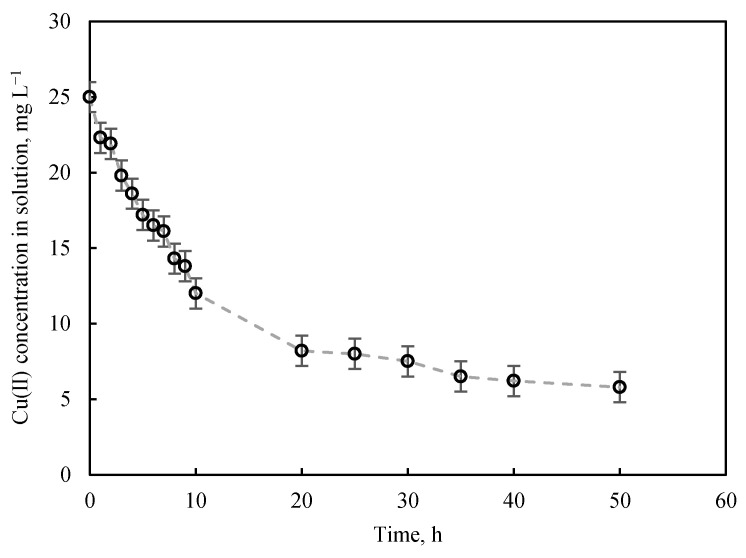
Adsorption kinetics of Cu (II) on marble waste sludges (C_0_ = 25 mg L^−1^, 0.1 g of adsorbent, V = 0.1 L at 25 °C, pH = 6). The dashed line is added to assist visualization.

Figure 1 depicts the Cu (II) adsorption curve for the marble waste sludges in ultrapure water, showing that the Cu (II) present is virtually eliminated after 50 h of contact.

The main stages in the adsorption kinetics are: transfer of the external material of the adsorbate (Cu) from the solution to the immediate adsorbent surroundings (marble waste sludges); internal diffusion of the adsorbate towards the pores of the adsorbent; and adsorption as such [34]. In the present experiment, external material transfer can be considered negligible because all systems were in constant agitation.

Different kinetic models were applied to quantify the Cu (II) adsorption rate on the sludge and to identify the chemical and textural properties involved. Pseudo-first [35] and pseudo-second [36] order kinetic models were the most widely applied, represented by Equations (3) and (4), respectively:

(3) $$Q_{t}=Q_{e}\left(1-e^{-k_{1}t}\right)$$

(4) $$Q_{t}=\frac{k_{2}Q_{e}^{2}t}{1+k_{2}Q_{e}t}$$

where *Q_t_* is the adsorption capacity at time *t* in mg g^−1^; *Q_e_* is the adsorption capacity at equilibrium; *k*_1_ and *k*_2_ are the pseudo-first and pseudo-second order kinetic constants in h^−1^ and h mg^−1^ h^−1^, respectively. Expressions 3 and 4 were linearized for the experimental fitting of data and to obtain the kinetic parameters corresponding to pseudo-first (Equation (5)) and pseudo-second (Equation (6)) order models:

(5) $$\log\left(Q_{e}-Q_{t}\right)=\log\left(Q_{e}\right)-\left(\frac{k_{1}}{2.303}\right)t$$

(6) $$\frac{t}{Q_{t}}=\frac{1}{k_{2}Q_{e}^{2}}+\frac{t}{Q_{e}}\;$$

Linear regression of $\log\left(Q_{e}-Q_{t}\right)$ versus *t* was used to obtain the pseudo-first order kinetic constant (*k*_1_) and its estimation of the adsorption capacity at equilibrium (*Q_e_*). Similarly, linear regression of *t Q_t_*^−1^ versus *t* was used to determine the pseudo-second order kinetic constant (*k*_2_) and *Q_e_* (Table 3).

According to the Elovich model [37], frequently used to describe chemisorption processes, the adsorption rate decreases exponentially with coverage of the adsorbent surface by the adsorbate. This model is described in its general form by Equation (7) and in its linear form by Equation (8):

(7) $$\frac{\partial Q_{t}}{\partial t}=\alpha \exp\left(-\beta Q_{t}\right)$$

(8) $$Q_{t}=\left(\frac{1}{\beta }\right)\ln\left(\alpha \beta \right)+\left(\frac{1}{\beta }\right)$$

where α is the initial adsorption rate in mg g^−1^ h^−1^, and β is a constant related to adsorbent coverage in g mg^−1^. Plotting *Q_t_* versus ln(*t*) yields a linear graph for the determination of α and β (Table 3).

**Figure 2:**
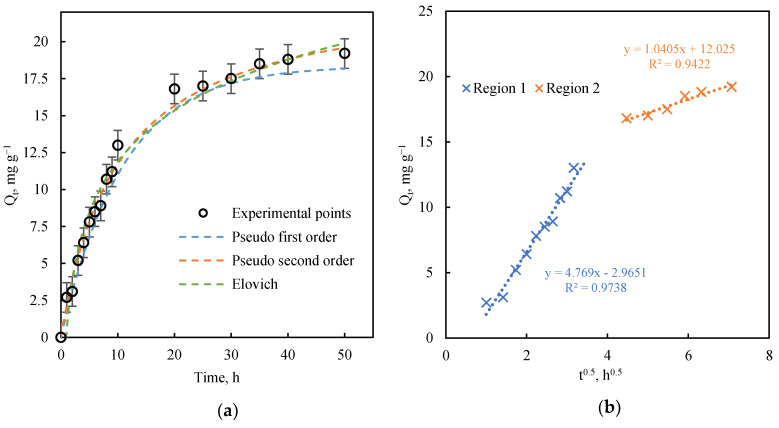
(**a**): Experimental kinetic curve and fitted kinetic models and (**b**): intraparticle diffusion model regression (C_0_ = 25 mg L^−1^, 0.1 g of adsorbent, V = 0.1 L at 25 °C, pH = 6).

The pseudo-second order kinetic model showed a better fit to the experimental data, with an R^2^ value of 0.992. Elevated R^2^ values were also obtained for the pseudo-first order and Elovich models, but they fitted the data less well. Figure 2a depicts the kinetic curves derived from each model with the experimental data, showing the better fit obtained with the pseudo-second order kinetic model.

The intraparticle diffusion kinetic model described by Weber and Morris [38] was used for the mechanistic study of Cu (II) diffusion in marble waste sludge, considering film diffusion, pore diffusion, and inter-particle diffusion to determine the controlling stage during adsorption. The general and linearized expressions of this model are described by Equations (9) and (10), respectively.

(9) $$Q_{t}=k_{d}\sqrt{t}\;$$

(10) $$Q_{t}=k_{d}\;t^{0.5}+C$$

where *k_d_* is the intraparticle diffusion rate constant in mg g^−1^ h^−0.5^, and *C* the so-called boundary level constant in mg g^−1^, which are determined by linear regression of *Q_t_* vs. *t*^0.5^ (Table 3). Figure 2b depicts two well-differentiated stages. The first stage was characterized by faster adsorption with a very low deviation with respect to the origin, suggesting that intraparticle diffusion is the controlling adsorption stage with no (or negligible) external material stage (or transport of dissolved Cu (II) towards the surface of the material). As noted above, this was expected because solutions were in constant agitation during the whole adsorption process. The second stage was appreciably slower because it was reaching equilibrium with a lower concentration of dissolved Cu (II). The value of *C* (boundary layer) in the second stage (12.025 mg g^−1^) was markedly higher than in the first, in which boundary layer effects were negligible (negative value) (Table 3). The boundary layer effect contributed to increasing resistance against Cu (II) ion transfer towards the adsorbent, with a negative effect on the adsorption rate. In parallel, the rapid initial phase is associated with the adsorption reaction, whereas the slow uptake at relatively high concentrations is usually associated with precipitation on the calcite surface, as observed by other researchers [23,39,40].

#### 3.2.2. Equilibrium Study

The study of adsorption equilibrium, specifically, the modeling of adsorption isotherms, is essential to study adsorbate–adsorbent affinity, its physicochemical properties, and the relationship between pollutant concentrations in equilibrium and the adsorptive capacities of a given material. Some of the most widely used models were applied in the equilibrium study.

The Langmuir model is used to describe monolayer adsorption on a homogeneous surface where there is no interaction among adsorbed molecules [5]. The Langmuir isotherm is represented by Equation (11).

(11) $$Q_{e}=\frac{Q_{m}K_{L}C_{e}}{1+K_{L}C_{e}}$$

where *K_L_* is the Langmuir equilibrium constant in L mg^−1^; *C_e_* is the concentration at adsorption equilibrium in mg L^−1^; *Q_m_* is the maximum adsorption capacity in mg g^−1^; and *Q_e_* is the adsorption capacity at equilibrium in mg g^−1^.

The Freundlich isotherm is used to describe multi-layer adsorption on a heterogeneous adsorbent surface with interactions among adsorbate molecules [11]. This model is represented by Equation (12).

(12) $$Q_{e}=K_{f}C_{e}{}^{1\;\cdot \;n^{-1}}\;$$

where *Q_e_* is the adsorption capacity at equilibrium in mg g^−1^; *K_f_* is the Freundlich constant related to adsorption capacity in mg g^−1^; *C_e_* is the concentration in equilibrium in mg L^−1^; and *n* is a parameter that represents the energetic heterogeneity of adsorption sites, considering adsorption as favorable when *n* > 1 and unfavorable when *n* < 1.

The Temkin isotherm considers adsorbent–adsorbate interactions and assumes that the adsorption heat decreases linearly with increased coverage of the surface. It is characterized by a uniform bond energy distribution until a maximum of bond energies is reached [41]. The model is represented by Equation (13):

(13) $$Q_{e}=B\;Ln\left(A_{T}C_{e}\right)$$

where *Q_e_* is the adsorption capacity at equilibrium in mg g^−1^; *C_e_* is the concentration at equilibrium in mg L^−1^; *B* is the adsorption heat-related constant of the model in J mol^−1^; and *A_T_* is the equilibrium binding constant in L mg^−1^.

The Dubinin–Radushkevich isotherm model predicts adsorptions on both homogeneous and heterogeneous surfaces by a Gaussian energy distribution mechanism [41]. It is represented by Equations (14) and (15):

(14) $$Q_{e}=Q_{m}e^{-K_{ad}\varepsilon^{2}}$$

(15) $$\varepsilon =RTLn\left(1+\frac{1}{C_{e}}\right)$$

where *Q_e_*, *Q_m_*, and *C_e_* are the same magnitudes as in the above models; *K_ad_* is the constant of the Dubinin–Radushkevich model in mol^2^ kJ^−2^; ε is the Polanyi potential; *R* is the gas constant, 8.314 J mol^−1^ K^−1^; and *T* is the temperature in K.

**Figure 3:**
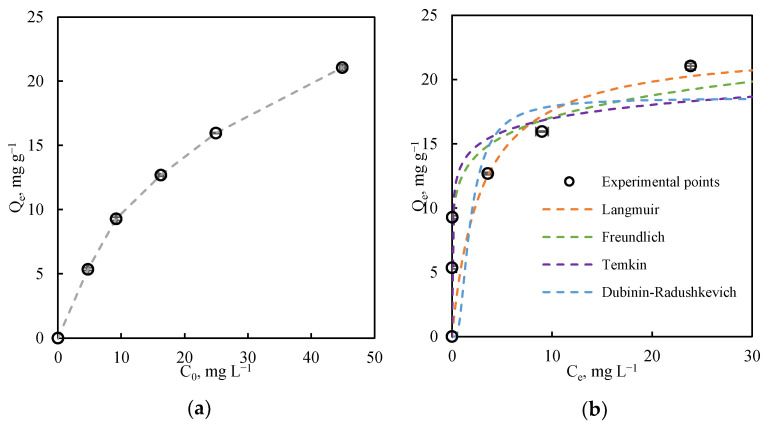
(**a**): Effect of initial Cu (II) concentration on adsorption capacities of waste marble sludges and (**b**): experimental adsorption isotherms and isotherm model plots for the removal of Cu (II) in ultrapure water by waste marble sludges (25 °C, pH = 6).

The characteristic parameters of each model were determined by non-linear fitting with the Excel Solver tool, applying the least squares error method between experimentally measured adsorption capacities and those estimated for each model. Figure 3a depicts the effect of initial Cu (II) concentrations on the adsorption capacity in equilibrium for the adsorbent under study. The curve does not reach stabilization, suggesting that the adsorption capacity of the marble sludges continues to increase with higher Cu (II) concentrations. Although the concentration range studied is low (in the ppm range), the adsorption capacity of this material is exceptional.

Table 4 lists the parameters obtained for each model. Figure 3b depicts the experimental points and plots of the isotherm models used. As observed in Figure 3b and based on the R^2^ values in Table 4 the Freundlich model provides the best fit to the experimental data, followed by the Langmuir, Temkin, and Dubinin–Radushkevich models. The value of n estimated by the Freundlich model is 7.3, much higher than 1, suggesting a very favorable adsorption of Cu (II) ions on the studied marble waste sludges.

The maximum adsorption capacity estimated by the Langmuir model is 22.74 mg g^−1^ for an initial Cu (II) concentration of 45 mg mL^−1^ at 25 °C, a higher value than found for Cu (II) adsorption on other materials (Table 5), indicating the potential usefulness of this marble waste sludge as a heavy metal adsorbent. Comparison of these experimental adsorption results with published data confirms that marble sludge can be an effective adsorbent for heavy metal removal from waters. Removal efficiencies obtained are similar to those observed with other industrial effluent treatment processes, such as ion exchange or osmosis [7].

### 3.3. Adsorption Mechanism

The characteristic reactions of metals with calcium oxide (predominant phase in marble waste sludges) explain the adsorption mechanism involved in the present experiments. Adsorption takes place at low concentrations of the metal in aqueous medium, whereas precipitation is predominant at high concentrations [39]. The adsorption of heavy metals on calcite is a complex process; however, it can be generally stated that: (i) metal cations with an ionic radius close to the Ca radius are more strongly adsorbed than are other metal cations and (ii) metals that form insoluble carbonates are adsorbed faster than those that form soluble carbonates.

**Figure 4:**
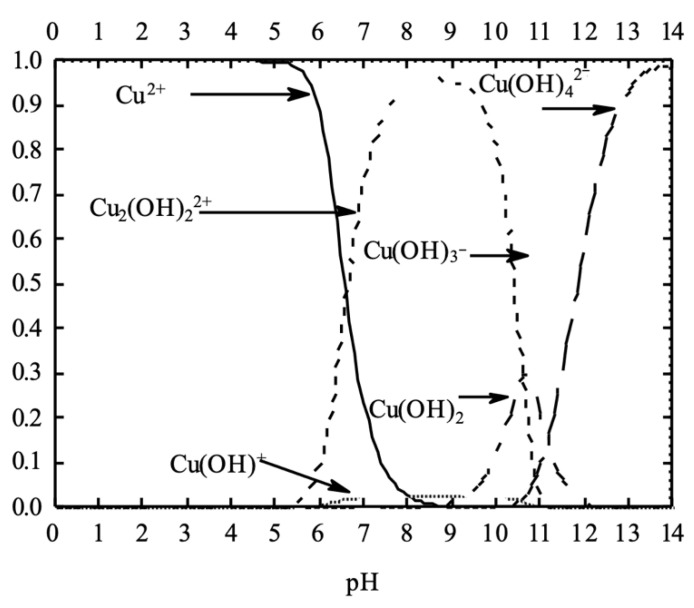
Cu (II) species distribution diagram as a function of pH.

According to the Cu (II) species distribution diagram as a function of pH (Figure 4), the hydrodynamic radius of the aquo-complex [Cu(H_2_O)_6_]^2+^, the predominant species at pH 6, is 0.069 nm [51] and the solubility product of CuCO_3_ is Kps = 2.5 × 10^−10^ [52]. The radius of Ca^2+^ is 0.099 nm [53].

Hence, Cu (II) adsorption on marble sludges may be produced by: (i) intercalation of the metal cation between CaCO_3_ layers and (ii) the formation of surface carbonate complexes.

**Figure 5:**
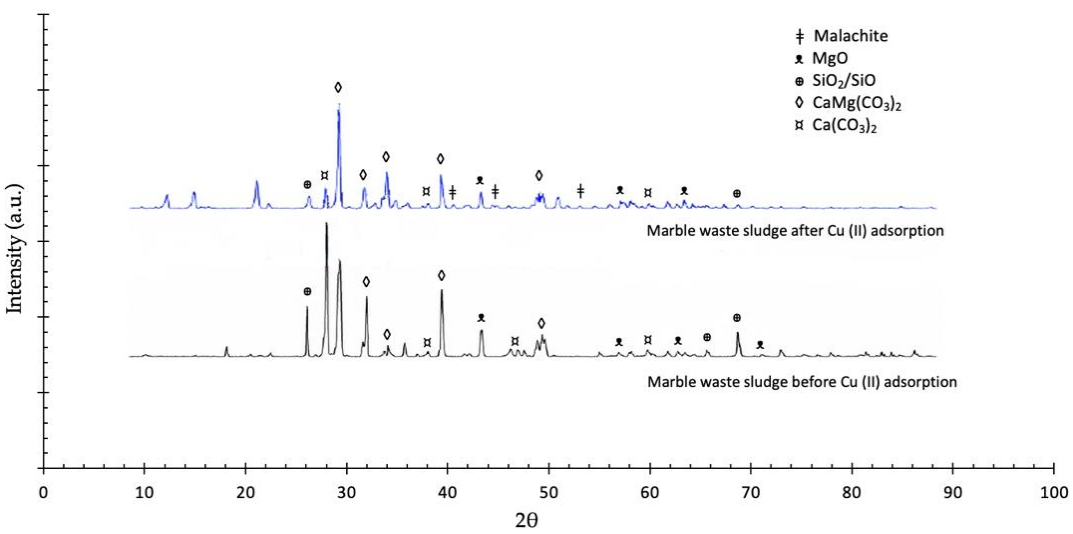
XRD of marble waste sludges before and after Cu (II) ion adsorption.

The XRD results for all marble waste sludge samples showed calcite (CaCO_3_) as the main phase. The mechanism involved in the adsorption process was identified by obtaining the XRD spectra of the waste sludge samples before and after Cu (II) adsorption. These reveal the formation of Cu (II) hydroxycarbonate (Figure 5), specifically malachite (Cu_2_(CO_3_)(OH)_2_), according to the characteristic diffraction peaks at 2θ = 37.7°, 39.4°, 42.4°, 43.4°, 46.1°, and 56.2° [54]. In addition, the formation of Cu (II) carbonate (CuCO_3_) after the adsorption process was observed as its characteristic diffraction peaks, located at 2θ = 12.1°, 14.3°, 21.0°, 32.9°, 34.3°, 36.0°, 40.0° and 50.2°, aroused in the spectrum in Figure 5 [55]. There are evident changes in the composition of the crystalline phase after metal ion adsorption with the appearance of some new peaks in the marble waste sludge diffractograms, as well as a general decrease in the peaks’ intensity, indicating the formation of a less ordered and crystalline structure. In addition, the calcite peak widths are not modified, indicating that Cu (II) adsorption on the sludge does not take place by intercalation between CaCO_3_ layers.

### 3.4. Influence of Operational Variables

#### 3.4.1. Influence of pH

**Figure 6:**
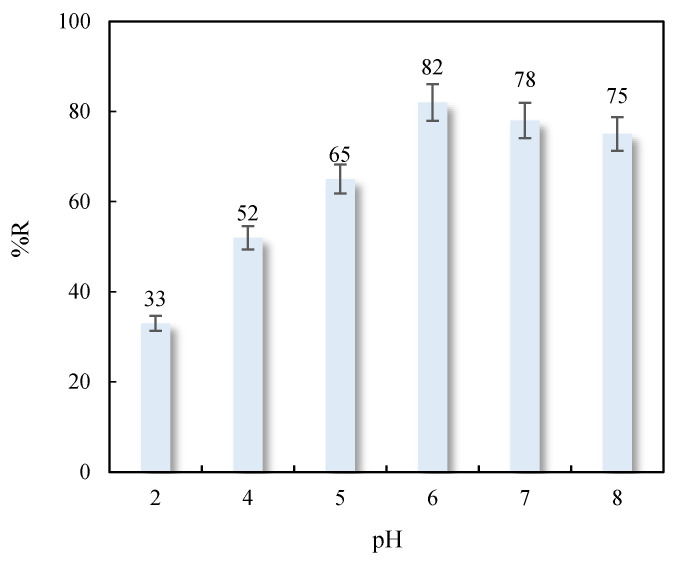
Influence of pH value on Cu (II) adsorption on marble waste sludges (25 °C, C_0_ = 25 mg L^−1^, 0.1 g of adsorbent).

Given the nature of the adsorbate and adsorbents used, the solution pH can affect Cu (II) adsorption on the carbonated sludges. Figure 6 depicts the results obtained by plotting the Cu (II) adsorption percentage against the solution pH, showing that the pH of the medium has a major impact on the adsorption process. Thus, the adsorption capacity increased with higher solution pH and reached an optimal value at pH values close to 6. This behavior can be explained by two factors: (i) a decrease in CuCO_3_ solubility at higher pH, favoring its precipitation on CaCO_3_, and (ii) electrostatic interactions between the Cu (II) aquo-complex and sludge surface. The pH of point of zero charge for carbonated sludges was close to 6 for all studied samples, indicating that the sludge surface was positively charged at pH < 6. The results in Figure 6 show a decrease in the negative charge of the aquo-complex at higher pH values, reducing attractive electrostatic attractions between sludge surface and aquo-complex. The adsorption process was not studied at pH values > 8 when there was complete precipitation of Cu (OH)_2_.

#### 3.4.2. Influence of Temperature

**Figure 7:**
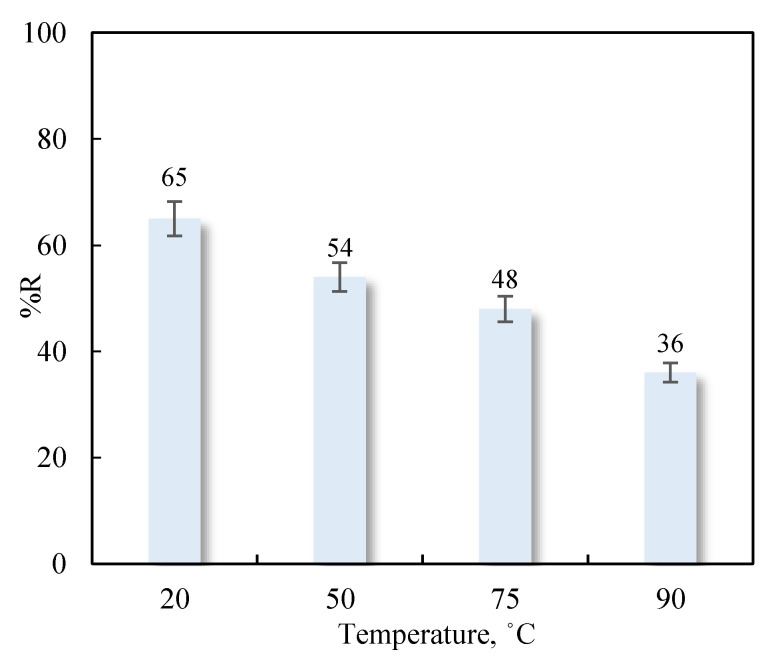
Influence of temperature on Cu (II) adsorption on marble waste sludges (pH = 5; C_0_ = 25 mg L^−1^, 0.1 g of adsorbent).

The adsorption of Cu (II) on carbonated sludges was studied at 20, 50, 75, and 90 °C (Figure 7). Results obtained revealed a marked decrease in percentage Cu (II) removal at higher temperatures, largely attributable to an increase in copper ion solubility, showing the same tendency as similar studies from literature [56,57]. This decrease in adsorption capacity may also indicate that Cu (II) adsorption is an exothermal process.

#### 3.4.3. Influence of Ionic Strength

**Figure 8:**
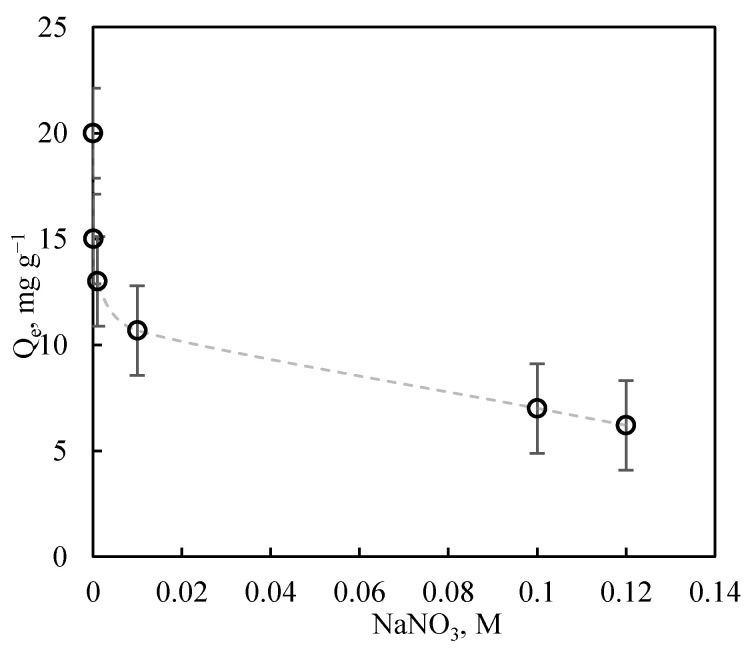
Influence of ionic strength on Cu (II) adsorption on sludges (pH = 5; 25 °C; C_0_ = 25 mg L^−1^, 0.1 g of adsorbent).

The ionic strength of solutions can have a greater or lesser impact on the adsorption of pollutants on adsorbent solids. The presence of electrolytes in solution has been reported to modify the strength of electrostatic adsorbate–adsorbent interactions [58,59]. Variations in the ionic strength of solutions can increase or decrease either attractive or repulsive interactions. Figure 8 depicts the exponential reduction in the adsorption capacity of Cu (II) with higher ionic strength, which can mainly be attributed to: (i) the increased solubility of CuCO_3_ and (ii) screening of the sludge surface by NO_3_^−^ anions, reducing attractive electrostatic interactions between the surface of the material and the Cu (II) aquo-complex.

#### 3.4.4. Influence of Chemical Composition of Water

A key issue is the effect of the chemical composition of the water on the efficiency of the process. The marble waste sludges evidenced a lower maximum Cu (II) adsorption capacity in surface waters, wastewaters, and groundwaters (see composition in Table 6) than in ultrapure water. This can be explained by: (i) the increased solubility of Cu (II) hydroxycarbonate due to the higher salinity of waters and (ii) the smaller surface area available for interaction between Cu (II) and the sludge due to the adsorption of dissolved organic matter.

The inactivation of nanomaterials by the presence of dissolved organic matter in the water media has been observed in other investigations using activated carbons as adsorbents [60]. In this study, the concentration of organic matter and water pH had a direct influence in the inactivation of marble waste sludges by organic matter. The concentration of organic matter and Cu (II) are in the same order of magnitude (mg/L), promoting a competition for the available sorption sites in the adsorbent. Molecular sizes of organic matter are two orders of magnitude larger [61] than the pore sizes of marble waste sludges (Table S1), facilitating pore blockage. That was especially noticeable for wastewaters, where the concentration of organic matter was the highest. On the other hand, surface waters possessed the highest pH in this study, leading to the lowest organic matter sorption (highest Cu (II) adsorption), mostly due to the repulsive forces generated between the adsorbent (negatively charged at pH > 6) and the organic matter.

### 3.5. Dynamic Adsorption Tests

The results (Table 7) obtained in a dynamic regime with the different types of water show that the amount adsorbed at the column breakthrough point (X_0.02_) was much lower than the adsorption capacity observed in static regime and was influenced by the type of water. According to these findings, the adsorption process is less effective in a dynamic regime than in a static regime because of the lesser Cu (II) diffusion into the interior of the CaCO_3_ sheets and the shorter contact time between adsorbate and adsorbent. The characteristics of the columns varied as a function of the chemical composition of the water. Thus, the lowest X_0.02_, V_0.02_ (breakthrough volume) and Du (degree of utility) values were obtained in wastewater. This may be attributable to the adsorption of organic matter on the surface of the material, which makes it difficult for Cu (II) to reaching active adsorption sites in an effective manner.

## 4. Conclusions

In this study, all sludges derived from marble cutting processes had the same chemical composition and surface characteristics, regardless of sample type. They were largely composed of calcite, and their surface area (by BET) ranged between 2 and 8 m^2^ g^−1^. This material had very low granulometry, where approximately 70% of the samples did not exceed the maximum size of 1 micron.

The results obtained for Cu (II) adsorption on marble waste sludges show that these materials can be an alternative option to the adsorbents most frequently used to remove heavy metals from waters (activated carbon and ion exchange resins). They have an adsorption capacity of around 21 mg g^−1^ at a Cu (II) concentration of 45 ppm at room temperature (25 °C). XRD analyses confirm that Cu (II) is adsorbed on marble waste sludges by precipitation in the form of Cu (II) hydroxycarbonate, specifically in the form of malachite.

Kinetics of Cu (II) adsorption followed a pseudo-second order model (k_2_ = 0.004 g mg^−1^ h^−1^). A mechanistic study of Cu (II) diffusion in marble waste sludge revealed two well differentiated stages: a first and rapid adsorption stage (with negligible mass transfer resistance of Cu (II) towards the surface of the material) related to the adsorption reaction; and a second and slower stage because of reaching equilibrium, boundary layer effects and precipitation of the adsorbate on the adsorbent surface.

Study of the influence of operational variables on the Cu (II) adsorption showed that the precipitation of Cu (II) on the sludge surface was reduced at higher temperature and water salinity values. The optimal pH value for the precipitation process was around 6.

Cu (II) removal is less effective from natural waters (surface waters, wastewaters, and groundwaters) for two reasons: (i) their elevated salinity increases the solubility of Cu (II) hydroxycarbonate, and (ii) a lesser surface area is available for interaction between Cu (II) and sludges due to the adsorption of dissolved organic matter.

The adsorption process is less effective in dynamic versus static regime due to lesser Cu (II) diffusion between the sheets of CaCO_3_ and shorter adsorbate–adsorbent contact time.

In conclusion, waste sludges from the marble industry potentially offer an effective and low-cost adsorbent for the removal of heavy metals such as Cu (II) from wastewaters. This is in line with new research on the application of carbonated waste, which forms a metal and calcium carbonate compound after heavy metal adsorption to remove proteins present in wastewaters.

## Figures and Tables

**Table 1:** Literature review of alternative adsorbents derived from marble production.

Adsorbent	Adsorbates	Performance	Ref.
Residual marble powder	Pb (II), Cu (II), Cd (II)	24.695, 19.4675, and 7.91 mg g^−1^ 90 min, pH 5–6	[22]
Marble powder mixtures	Cu, Zn, Mn, Zr	Removed 51–96% of heavy metals	[23]
Marble sludge	Cd, Cu, Pb, Zn	0.030, 0.53, 0.022 and 0.36 mg g^−1^	[29]
Zeolite synthesized from marble powder	Zn, Ni, Pb, Cr, Cd, Cu	Removed 75–99% of heavy metals	[24]
Marble powder	F^−^	1.20 mg g^−1^, pH = 7	[30]
Marble powder	Acid mine drainage Cd, Cr, Cu, Ni, Pb, Zn	Removed 80% of heavy metals	[25]
Marble powder waste	Cd (II)	Removed 99.45% of Cd (II) 20 mg L^−1^, pH = 7	[31]
Crushed marble	F^−^	0.7 mg L^−1^, 5 min, pH = 2, 25 °C	[32]

**Table 2:** Chemical composition of the waste sludges obtained by X-ray fluorescence. M1 to M6 represent successively each of the individual marble waste sludge samples.

Sludge Sample	SiO_2_ %	Al_2_O_3_ %	Fe_2_O_3_ %	MnO %	MgO %	CaO %	Na_2_O %	K_2_O %	TiO_2_ %	P_2_O_5_ %	LOI * %	Addition %
M1	0.04	<LLD	0.01	<LLD	0.32	55.52	0.07	0.01	<LLD	0.01	43.33	99.30
M2	0.21	0.02	0.06	0.01	1.03	55.33	0.06	0.02	<LLD	0.01	43.40	100.13
M3	0.28	0.06	0.20	0.02	1.12	54.95	0.07	0.03	<LLD	0.01	43.51	100.25
M4	0.28	0.06	0.36	0.03	0.84	55.15	0.07	0.03	<LLD	0.01	43.24	100.07
M5	0.27	0.06	0.21	0.03	1.07	54.92	0.07	0.02	<LLD	0.01	43.40	100.06
M6	0.25	0.05	0.05	0.01	1.01	55.27	0.07	0.03	<LLD	0.01	43.40	100.01

* Loss On Ignition (LOI): % of moisture and volatiles contained in the samples.

**Table 3:** Kinetic parameters for Cu (II) adsorption onto marble waste sludges.

	Model Parameters	R^2^
**Pseudo-first order**		
*k*_1_, h^−1^	0.091	0.987
*Q_e_*, mg g^−1^	18.361	
**Pseudo-second order**		
*k*_2_, g mg^−1^ h^−1^	0.004	0.992
*Q_e_*, mg g^−1^	23.529	
**Elovich**		
*α*, mg g^−1^ h^−1^	5.410	0.972
*β*, g mg^−1^	0.201	
**Intraparticle diffusion**	*First region*	
*k*, mg g^−1^ h^−0.5^	4.769	0.974
*C*, mg g^−1^	−2.965	
	*Second region*	
*k*, mg g^−1^ h^−0.5^	1.041	0.942
*C*, mg g^−1^	12.025	

**Table 4:** Isotherm parameters for the adsorption of Cu (II) onto waste marble powders.

	Model Parameters	R^2^
Langmuir		0.834
*K_L_*, L mg^−1^	0.342	
*Q_m_*, mg g^−1^	22.745	
Freundlich		0.909
*K_f_*, mg g^−1^	12.458	
*n*	7.308	
Temkin		0.779
*B*, J mol^−1^	1.539	
*A_T_*, L mg^−1^	6175.302	
Dubinin		0.689
*K_ad_*, mol^2^ kJ^−2^	6.35 × 10^−7^	
*Q_m_*, mg g^−1^	18.572	

**Table 5:** Adsorption of Cu (II) on inorganic materials.

Adsorbent	Q (mg g^−1^)	pH	Initial Concentration of Cu (II) (mg L^−1^)	Ref
Marble waste sludge	20–23	6	45	*This work*
Marble powder	222.84	6	2000	[5]
Egg shell	150	6	30,000	[42]
	5.03	6.5	100	[43]
Egg shell powder coated with iron oxide	44.84	6	100	[44]
Limestone	0.59	9	100	[29]
	0.0145	8.5	2	[40]
Dolomite	0.60	10	100	[29]
	8.26	-	2400	[45]
Iron oxide-vermiculite compound	59.70	5	500	[46]
Waste iron oxide	17.08	6	35	[47]
Ca-montmorillonite	9.86	-	164	[48]
Spartina alterniflora-derived biochar	49.14	6	290	[49]
Activated carbon derived from Tunisian date stones	31.25	5	100	[50]

**Table 6:** Composition of natural waters used in the study.

Type of Water	pH	[HCO^3−^], meq L^−1^	TOC, mg L^−1^	Q_m_, mg g^−1^
Ultrapure water	5.8	0.0	0.0	22.75
Surface water	8.3	6.4	11.9	12.4
Groundwater	7.5	8.8	10.3	8.65
Wastewater	7.8	7.2	17.0	6.75

**Table 7:** Results derived from the Cu (II) breakthrough curves in natural waters in waste sludge columns.

Type of Water	X_0.02_, mg g^−1^	V_0.02_, L	Φ	HMTZ, cm	Du, %
Ultrapure water	8.88	0.57	7.37	5.62	81.52
Surface water	7.06	0.45	2.33	6.43	81.53
Groundwater	5.06	0.32	5.14	7.56	80.80
Wastewater	3.34	0.21	9.35	8.64	17.34

X_0.02_: Amount adsorbed at breakthrough point; V_0.02_: Breakthrough volume; Φ: Fractional capacity; HMTZ: Height of mass transfer zone; Du: Degree of utility.

## Supplementary Materials

The following are available online at https://www.mdpi.com/article/10.3390/nano11092305/s1, Figure S1: SEM images of individual marble waste sludges (M1-M6), Figure S2: Granulometric analysis of marble waste sludges, Figure S3: (a): Effect of initial Cu (II) concentration in adsorption capacities of individual waste marble sludges samples and (b): individual adsorption isotherms for the removal of Cu (II) in ultrapure water by waste marble sludges (25 °C, pH = 6), Table S1: Textural properties of individual marble waste sludges samples.
