# Supplementary material for: Marble Waste Sludges as Effective Nanomaterials for Cu (II) Adsorption in Aqueous Media
Source: Nanomaterials (Basel). 2021 Sep 5;11(9):2305. doi: 10.3390/nano11092305 (PMC8466878; doi:10.3390/nano11092305)
Supplement: Supplementary file 1 [file nanomaterials-11-02305-s001.zip › nanomaterials-1352174-supplementary.pdf]

SUPPORTING INFORMATION (SI)

Marble Waste Sludges as Effective Nanomaterials for Cu (II) Adsorption in Aqueous Media

Ventura Castillo Ramos [1,*], José Rivera Utrilla [1], Antonio Ruiz Sánchez [2], María Victoria López Ramón [3] and Manuel Sánchez Polo[1]

[1] Affiliation 1; Department of Inorganic Chemistry, Faculty of Science, University of Granada, 18071, Granada, Spain. vcastillo@ugr.es, mansanch@ugr.es, jrivera@ugr.es
[2] Affiliation 2; Department of Structure Mechanics and Hydraulic Engineering, University of Granada, 18071, Granada, Spain. antonioruiz@ugr.es
[3] Affiliation 3; Department of Inorganic and Organic Chemistry, Faculty of Experimental Science, University of Jaén, 23071, Jaén, Spain. mvlro@ujaen.es
* Correspondence: vcastillo@ugr.es

1. SEM images, granulometric analysis and textural properties of marble waste sludges

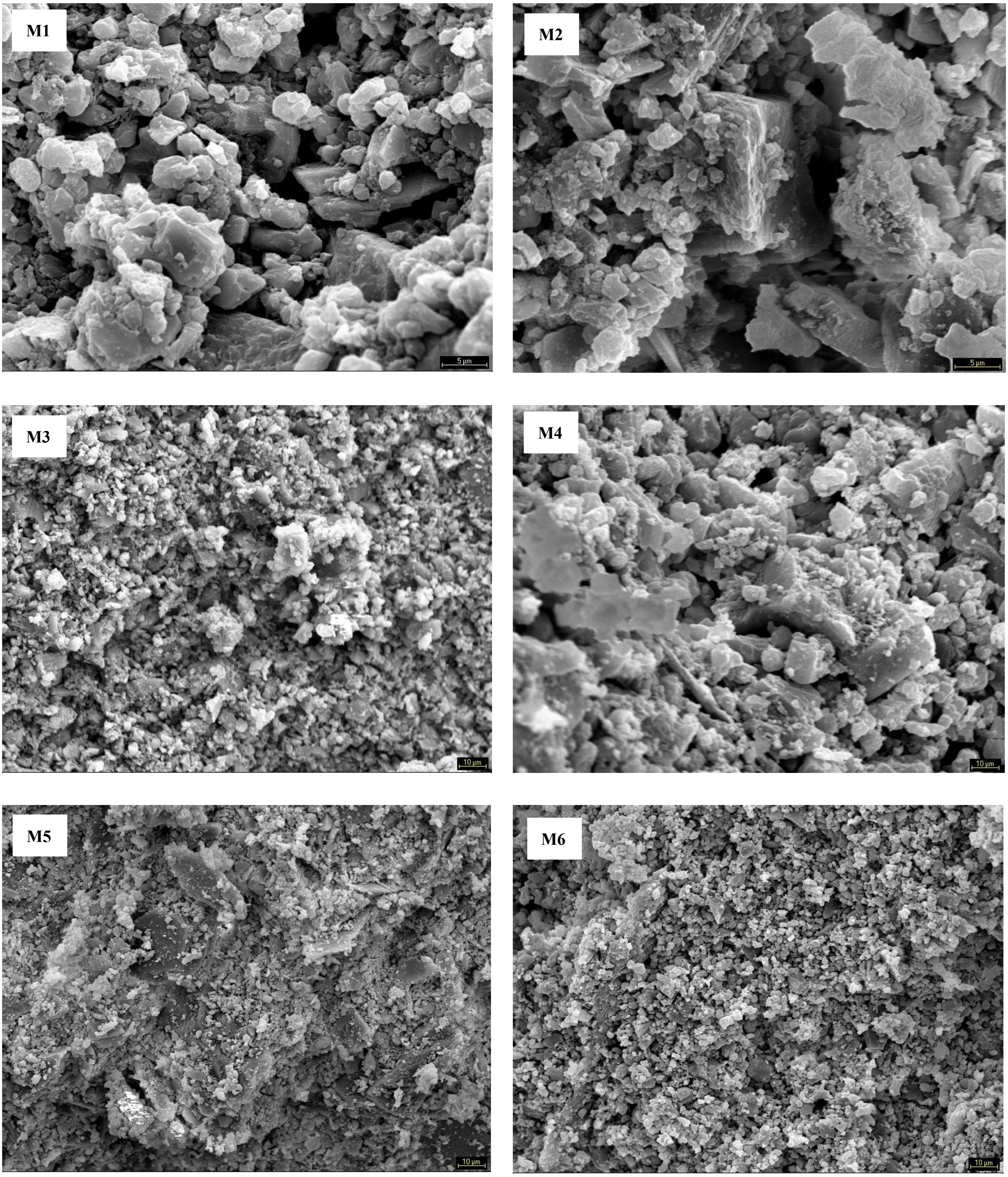

Figure S1. SEM images of marble waste sludges. M1 to M6 represent successively each of the individual marble sludge samples.

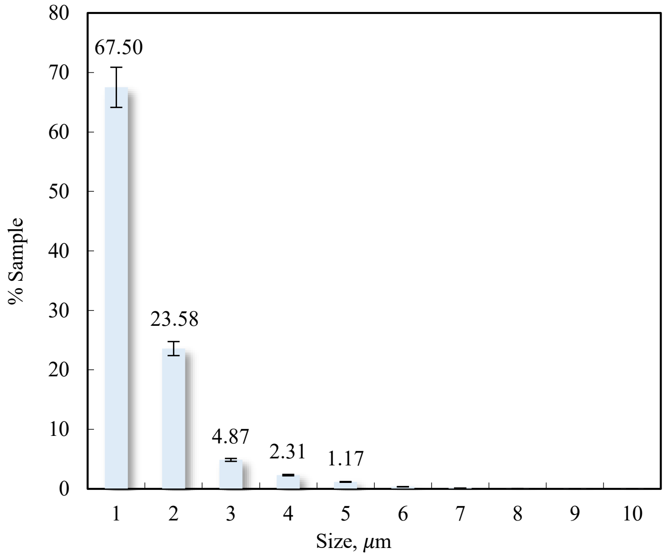

Figure S2. Granulometric analysis of marble waste sludges

N_2 adsorption isotherms were obtained in an AUTOSORB-1C volumetric instrument of Quantachrome Instruments, which can achieve a vacuum of 10^{-7} mbar by the combined action of a dry and a turbomolecular pump with liquid N_2 cold trap. Isotherms were obtained by introducing 0.1 g of sample dried at 383 K in a glass flask, placing it in the volumetric apparatus, and degasifying it at the same temperature overnight in dynamic vacuum of 10^{-6} mbar. N_2 adsorption isotherms at 77 K were used to determine the surface area (S_{BET}), micropore volume (W_0), and mean diameter (L) (Table S1). The results show that the sludges generated in the extraction process have a very low surface area (2–8 m^2/g) and micropore volume, with a mean pore diameter that ranges between 2 and 5 nm.

Table S1. Textural properties of individual marble waste sludges samples

Sample	S_{BET} (m^2/g)	V·10^3 (cc/g)	L (nm)
M1	8.9	1.15	3.34
M2	2.4	8.93	4.14
M3	1.7	1.72	2.29
M4	1.6	5.68	4.67
M5	5.1	2.84	5.62
M6	1.8	1.75	2.24

2. Individial adsorption isotherms of marble waste sludges

As observed in Figure S2, the adsorption capacities at each concentration (initial and equilibrium) of copper ions in water have a similar adsorption profile in all samples, suggesting that the chemical composition of the samples does not significantly interact in the adsorption process.

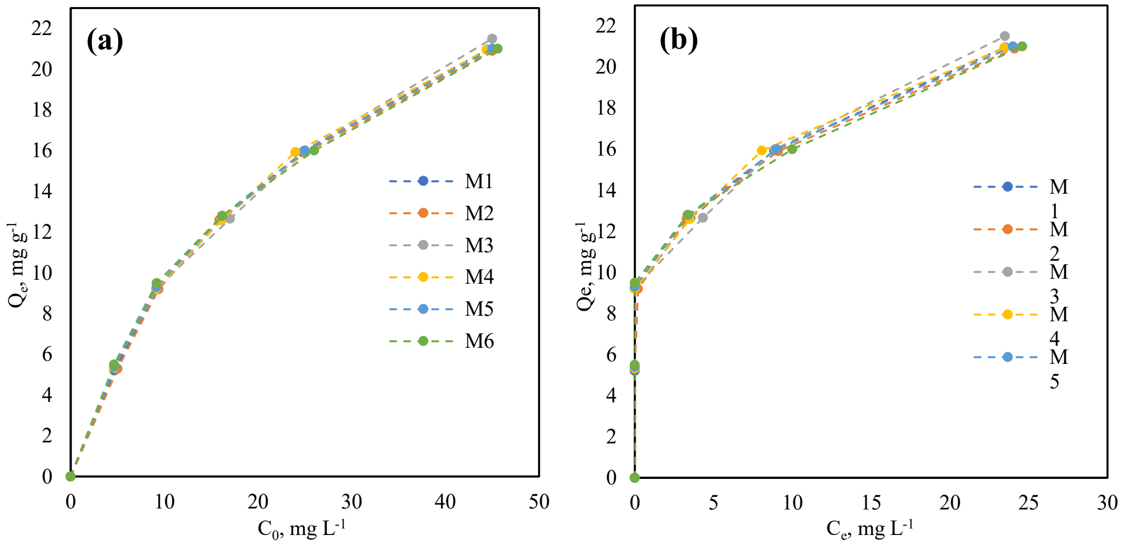

Figure S3. (a): Effect of inicial Cu (II) concentration in adsorption capacities of individual waste marble sludges samples and (b): individual adsorption isotherms for the removal of Cu (II) in ultrapure water by waste marble sludges (25°C, pH = 6).
